# Comparative Prognostic Performance of Platelet-Containing and Platelet-Free Inflammatory Indices After Percutaneous Coronary Intervention

**DOI:** 10.3390/jcm15176872

**Published:** 2026-09-04

**Authors:** Donghyeon Joo, Sungho Jo, Jeong Tae Byoun, Jae Young Cho, Kyeong Ho Yun

**Affiliations:** Department of Cardiovascular Medicine, Regional Cardiocerebrovascular Center, Wonkwang University Hospital, 895 Muwang-ro, Iksan 54538, Republic of Korea; dhjoozz@naver.com (D.J.); fuenrostu@naver.com (S.J.); overchaos12@gmail.com (J.T.B.); librato46@gmail.com (J.Y.C.)

**Keywords:** percutaneous coronary intervention, systemic immune–inflammation index, pan-immune–inflammation value, neutrophil-to-lymphocyte ratio, blood platelets, inflammation, risk stratification, landmark analysis

## Abstract

**Background/Objectives:** Systemic inflammatory indices derived from the complete blood count differ in whether they incorporate platelet count. Whether platelet-containing indices outperform platelet-free indices and whether platelet count adds independent prognostic information remain insufficiently tested. **Methods:** We retrospectively analyzed 2244 consecutive patients undergoing percutaneous coronary intervention (PCI) at a single center. The neutrophil-to-lymphocyte ratio (NLR), systemic inflammatory response index (SIRI), systemic immune–inflammation index (SII) and pan-immune–inflammation value (PIV) were derived from the baseline blood count. The outcome was 1-year (400-day) major adverse cardiovascular events (MACEs). Because SII and PIV equal NLR and SIRI multiplied by platelet count, the platelet contribution was tested with nested likelihood ratio tests. Calibration, decision-curve, 30-day landmark, time-varying coefficient and subgroup analyses were also performed. **Results:** MACEs occurred in 170 patients (7.6%). After adjustment, NLR, SII and PIV remained associated with MACEs. SII showed the highest C-index and lowest AIC, but no index significantly improved discrimination over the clinical model (ΔC-index +0.009, *p* = 0.060 for SII), no head-to-head difference was detected, and the added net benefit was negligible (0.38 per 100 patients). Adding platelet count did not improve model fit over the whole follow-up (*p* = 0.104 and 0.146) but did beyond the 30-day landmark (*p* = 0.025 and 0.033); the platelet-by-period interaction was not significant (*p* = 0.197). Exploratory analyses suggested larger associations in ST-segment elevation MI based on few events. Only the adjusted SII association survived within-family Holm correction; no result met the global 5% false-discovery-rate threshold. **Conclusions:** The four indices showed similar, modest discrimination without evidence of superiority or meaningful incremental utility; the post-30-day platelet signal was not confirmed by interaction testing and remains hypothesis-generating.

## 1. Introduction

Inflammation contributes to the initiation, progression and clinical destabilization of coronary artery disease, and inflammatory activation remains prognostically relevant in patients undergoing percutaneous coronary intervention (PCI). Platelets participate in this process not only through thrombus formation but also through interactions with leukocytes and endothelial cells that couple inflammation to vascular injury [1]. Consequently, inflammatory indices derived from the routine complete blood count have attracted attention as inexpensive and universally available tools for risk stratification after PCI.

The neutrophil-to-lymphocyte ratio (NLR) is the most extensively studied of these indices and has been associated with adverse outcomes in patients with myocardial infarction (MI) undergoing PCI [2]. Composite indices incorporating additional cell lineages have subsequently been proposed. The systemic inflammatory response index (SIRI) reflects the combined balance of innate inflammatory activation and lymphocyte-mediated immune regulation and predicts adverse events in acute coronary syndrome (ACS) treated with PCI [3]. The systemic immune–inflammation index (SII) has been associated with outcomes in coronary artery disease and after PCI [4,5], and the pan-immune–inflammation value (PIV) has more recently been evaluated in ACS [6]. These indices fall naturally into two groups: platelet-free indices, comprising NLR and SIRI, and platelet-containing indices, comprising SII and PIV.

Head-to-head comparisons among these indices remain scarce. Ting et al. compared six complete blood count-derived markers, including NLR, SIRI, SII and PIV, for one-year mortality and reinfarction in 689 patients with ST-segment elevation MI, and reported that several were independently associated with mortality after adjustment [7]. That analysis, however, dichotomized each marker at a receiver operating characteristic-derived cut-off, did not quantify incremental value beyond a clinical model using formal model-performance metrics, and did not adjust for a circulating inflammatory biomarker such as high-sensitivity C-reactive protein (hsCRP). A recent systematic review and meta-analysis reached a similar conclusion for PIV specifically, noting that whether it confers incremental discrimination, calibration or reclassification beyond NLR, SII or SIRI remains unproven, and called for adequately powered studies that pre-specify direct comparisons of multiple indices within a single cohort [8].

Two features of these indices make such a comparison tractable. First, because SII is the product of NLR and platelet count and PIV is the product of SIRI and platelet count, the question of whether platelet-containing indices outperform their platelet-free counterparts reduces, on the logarithmic scale, to whether platelet count carries prognostic information independent of the leukocyte-derived ratio—a single-degree-of-freedom comparison between nested models. Second, whether any of these indices retains prognostic value beyond the early post-PCI phase is unclear, since periprocedural leukocyte and platelet responses may be driven by infarct severity, plaque instability and procedural injury rather than by durable inflammatory risk.

Therefore, we compared platelet-free and platelet-containing systemic inflammatory indices within a single all-comer PCI cohort. The study is positioned as a comparative, model-performance investigation rather than as an attempt to establish a new clinically actionable biomarker: the association of SII and PIV with cardiovascular outcomes is already well documented in meta-analyses [5,8]; what remains insufficiently tested is whether their incremental predictive performance differs within the same cohort and whether platelet count adds prognostic information beyond the leukocyte-derived component. The objectives were to determine whether each index is independently associated with 1-year major adverse cardiovascular events (MACEs) after multivariable adjustment; to quantify and formally compare the incremental value of each index beyond a clinical model, with explicit nested comparisons isolating the contribution of platelet count; to assess calibration and clinical utility; and to assess whether any prognostic signal persists beyond the early post-PCI phase in a 30-day landmark analysis, supplemented by time-varying coefficient models and by subgroup analyses according to clinical presentation.

## 2. Materials and Methods

### 2.1. Study Population

**Figure 1:**
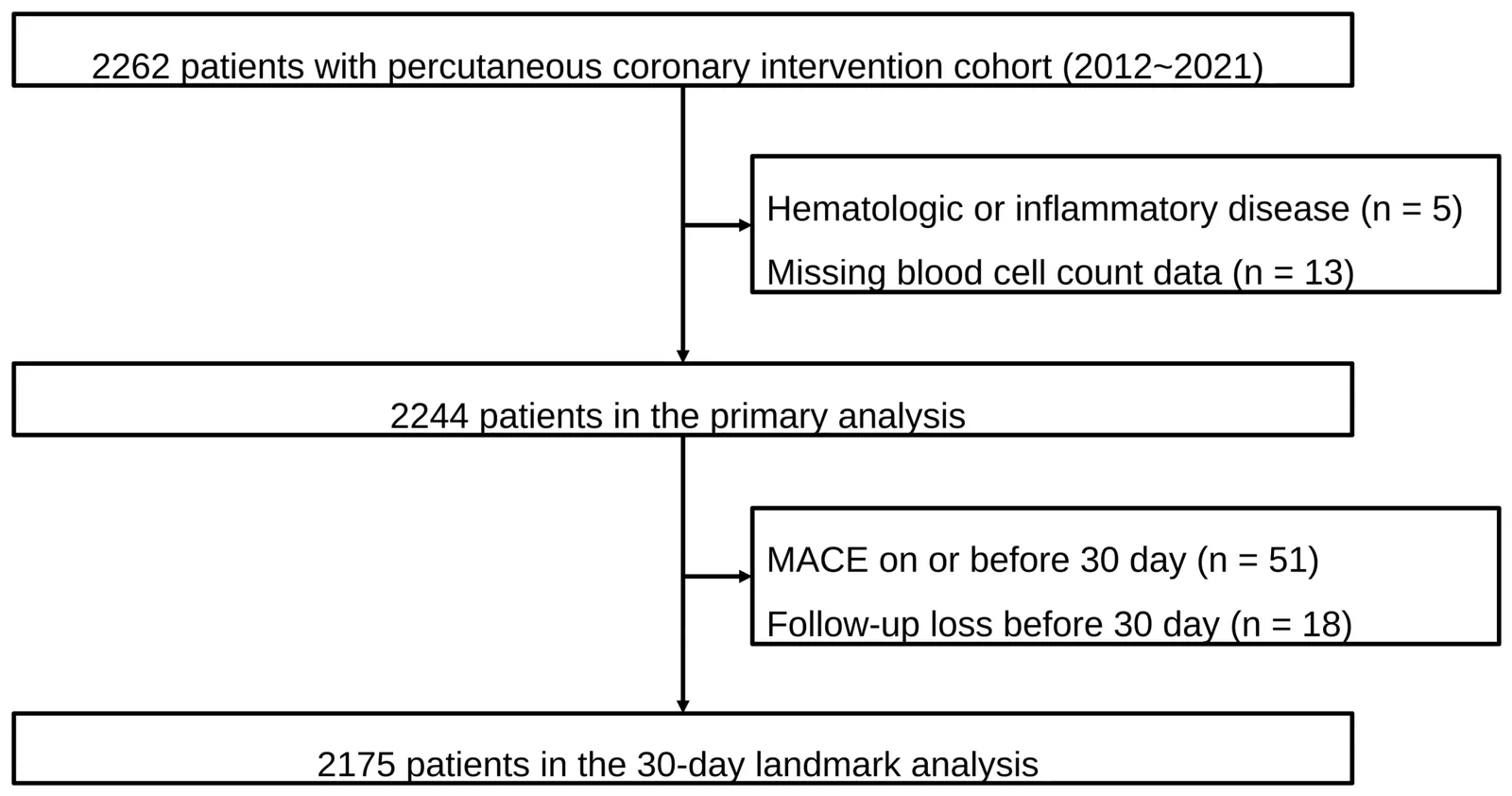
Study flow diagram. MACE, major adverse cardiovascular event.

This was a retrospective observational study based on a single tertiary-center PCI cohort. Between 2012 and 2021, 2262 consecutive patients who underwent PCI were identified. Thirteen patients with missing neutrophil, lymphocyte, monocyte or platelet counts and five patients with a hematologic or inflammatory disease were excluded, leaving 2244 patients for analysis (Figure 1).

The study protocol was approved by the Institutional Review Board of Wonkwang University Hospital (approval No. 2026-06-044), which granted retrospective approval for the analysis of previously collected data. The requirement for written informed consent was waived because of the retrospective observational design. The study was conducted in accordance with the Declaration of Helsinki.

### 2.2. Data Collection and Definitions

Clinical, angiographic, procedural and laboratory data were obtained from electronic medical records and the institutional PCI database. Baseline laboratory testing included the complete blood count, hemoglobin, creatinine, lipid profile and hsCRP. All blood samples were obtained in the emergency department or at admission, before coronary angiography and before administration of contrast, heparin or antiplatelet loading; the interval between symptom onset and sampling was not recorded. The estimated glomerular filtration rate (eGFR) was calculated with the 2021 race-free Chronic Kidney Disease Epidemiology Collaboration creatinine equation [9]. Left ventricular ejection fraction (LVEF) was measured by transthoracic echocardiography during the index admission. Multivessel disease was defined as significant stenosis in two or more epicardial territories, with left main disease counted as two. Complex PCI was defined by the presence of at least one of the following: three or more treated lesions, three or more stents implanted, total stent length greater than 60 mm, a bifurcation lesion treated with two stents, or a chronic total occlusion [10].

### 2.3. Systemic Inflammatory Indices

Neutrophil, lymphocyte, monocyte and platelet counts obtained before coronary angiography for the index PCI were used. Leukocyte subpopulation counts are reported by the laboratory in cells/μL and were divided by 1000 before being entered into the formulae, so all four cell counts were expressed in the same unit of 10^3^/μL. The indices were calculated as NLR = neutrophil/lymphocyte [11]; SIRI = neutrophil × monocyte/lymphocyte [3]; SII = platelet × neutrophil/lymphocyte [4]; and PIV = platelet × neutrophil × monocyte/lymphocyte [6]. NLR and SIRI are platelet-free; SII and PIV are platelet-containing. On the logarithmic scale, the two families are related by log(SII) = log(NLR) + log(platelet) and log(PIV) = log(SIRI) + log(platelet). Throughout this report, log denotes the natural logarithm.

### 2.4. Outcomes

The outcome was MACE, defined as the first occurrence of all-cause death, MI, ischemic stroke or any revascularization. Follow-up began on the day of the index PCI and was administratively censored at 400 days. Clinical follow-up was scheduled at one year, and the 400-day limit was chosen so that events ascertained at a follow-up visit occurring within the permitted window after the anniversary date were captured. Outcomes are referred to throughout as 1-year outcomes and in every instance denote events occurring within 400 days of the index procedure. In 23 event records belonging to 12 patients, an event date had been recorded after the documented end of follow-up. In every case, both dates lay beyond day 400, so these discordant records fell outside the 400-day analysis window; no event date was reassigned, and the results are identical whether these records are retained as recorded or disregarded, because neither treatment affects the window. Eleven of the twelve patients were censored at day 400 and one had an earlier, concordant event within the window; the effect of excluding all 12 patients is reported in Section 3.9. Because the component events were not mutually exclusive, MACE was analyzed according to the first component to occur.

### 2.5. Statistical Analysis

Continuous variables are presented as mean ± standard deviation or median [interquartile range] according to their distribution, and categorical variables as numbers and percentages. Groups were compared with the Welch *t*-test, the Mann–Whitney *U* test or the chi-square test as appropriate.

All four indices were natural log-transformed because of their right-skewed distributions and then standardized, so hazard ratios (HRs) are expressed per one standard deviation (SD) increase. Time-to-event associations were assessed with Cox proportional hazards models. A single adjustment set was specified before analysis, comprising age, sex, hypertension, diabetes mellitus, MI presentation, hemoglobin, eGFR, log-transformed hsCRP, multivessel disease and LVEF—that is, the demographic, clinical, laboratory, angiographic and echocardiographic variables routinely available at the time of the index procedure. These covariates are referred to collectively as the clinical model. The proportional hazards assumption was examined with scaled Schoenfeld residuals (rank transformation of time). Departure from log-linearity was tested for each index within the adjusted model by comparing the log-linear specification with one in which the index was entered as a restricted cubic spline with four knots placed at the 5th, 35th, 65th and 95th percentiles.

Incremental predictive performance was quantified with Harrell’s C-index [12], the inverse-probability-of-censoring-weighted cumulative/dynamic area under the curve (AUC) [13] at the end of the 400-day follow-up window, the Akaike information criterion (AIC), the likelihood ratio test (LRT) against the nested clinical model, and an optimism-corrected C-index obtained by bootstrap internal validation with 1000 resamples. Because follow-up was administratively censored at exactly 400 days, the censoring survival function reaches zero at that instant and the censoring weights are undefined; the AUC was, therefore, evaluated at day 399, the last evaluable time point, which still includes every observed event (the latest event occurred on day 398). Differences in C-index—both between each marker-added model and the clinical model, and between marker-added models—were tested with a paired bootstrap in which both models were refitted within each 1000 resamples, with two-sided *p* values derived from the proportion of resampled differences crossing zero. Calibration was assessed by the bootstrap optimism-corrected calibration slope of the linear predictor (1000 resamples); by the observed-to-expected ratio, defined as the observed Kaplan–Meier risk at day 399 divided by the mean predicted risk, which was used as the fixed-horizon measure of calibration-in-the-large appropriate for censored survival data; and by comparing predicted with observed Kaplan–Meier risk at day 399 across deciles of predicted risk. Overall accuracy was quantified with the inverse-probability-of-censoring-weighted Brier score at day 399, the integrated Brier score over days 30–390, and the index of prediction accuracy (IPA), which scales the Brier score against a covariate-free Kaplan–Meier reference [14]. Clinical utility was evaluated by decision-curve analysis for survival data, expressing net benefit per 100 patients across threshold probabilities of 1-year MACEs from 1% to 30% [15].

To isolate the contribution of platelet count, standardized log-transformed platelet count was entered as a free parameter alongside the corresponding platelet-free index, and the resulting model was compared with the platelet-free model by a single-degree-of-freedom LRT. The same unconstrained model was then compared with the corresponding platelet-containing index model. Because log(SII) = log(NLR) + log(platelet) holds exactly, the index model lies within the space spanned by its two components, so this second comparison is also a proper single-degree-of-freedom test; on the untransformed logarithmic scale, it constrains the two component coefficients to be equal. The Cox partial likelihood is invariant to the centering and scaling of covariates, so these tests are unaffected by the standardization used to express hazard ratios per 1 SD, and the constrained model was fitted by entering log(SII) or log(PIV) itself rather than by imposing a constraint on the standardized components. All models within each comparison were fitted on an identical complete-case sample so that AIC values and likelihood ratio tests were directly comparable.

A 30-day landmark analysis was planned before the analyses reported here to assess whether the indices retain prognostic value beyond the early post-PCI phase. Patients who experienced MACEs on or before day 30 and those lost to follow-up before day 30 without an event were excluded; follow-up time was then measured from the landmark [16]. Because comparing two separate analyses does not in itself establish that an effect differs between periods, the difference was tested directly: the follow-up of every patient was split into episodes on or before and after day 30, and an interaction between log-transformed platelet count and period was estimated in a single model over the whole follow-up. To avoid reliance on a single landmark, time-varying coefficient models were also fitted in which follow-up was divided into four episodes (0–30, 31–90, 91–180 and 181–400 days) and a separate coefficient was estimated for each period, with heterogeneity tested by a likelihood ratio test. A linear trend in log(time + 1) was tested with the scaled Schoenfeld score test using a logarithmic time transform, and confirmed in a model in which follow-up was split at every distinct event time and the product of the covariate with log(time), evaluated at the start of each episode, was entered as a time-varying term, so that covariate values within a risk set depend only on the current time. Cumulative event curves were generated with the Kaplan–Meier method expressed as one minus the survival probability and compared with the log-rank test.

Heterogeneity by clinical presentation was examined in three ways: acute coronary syndrome versus chronic coronary syndrome; myocardial infarction versus no myocardial infarction; and ST-segment elevation MI versus non-ST-segment elevation MI versus no MI. Within each stratum, the adjusted model was refitted with the presentation term removed, and interaction was tested by adding index-by-presentation product terms to the fully adjusted model on the whole cohort. Adjusted models were fitted within strata, and for individual components of MACE, only when there were at least five events per model parameter (that is, at least 50 events for the 10-parameter models within presentation strata and at least 55 events for the 11-parameter component models); otherwise, univariable estimates are reported. Calendar era (2012–2016 versus 2017–2021) was examined as an additional covariate, as a stratification variable and in interaction with each index.

Additional sensitivity analyses censored follow-up at 365 days; examined each component of MACE separately; adjusted for troponin T, which had been excluded from the primary model because the assay changed from a conventional to a high-sensitivity assay in 2018, using van der Waerden normal scores computed within each assay era to place the two eras on a common percentile scale; and repeated the primary analysis after multiple imputation of missing covariates with chained equations (20 imputed datasets, pooled with Rubin’s rules). Because the analyses generate numerous inferential tests, multiplicity adjustment was applied to the 21 principal inferential tests, grouped into six families: the adjusted associations of the four indices with MACE; the four tests of the differences in C-index between each index-added model and the clinical model; the three head-to-head comparisons; the four likelihood ratio tests for adding platelet count, over the full period and within the landmark period; the four adjusted associations in the landmark analysis; and the two platelet-by-period interaction tests. Holm step-down adjustment was applied within each family, and Benjamini–Hochberg false-discovery-rate adjustment was applied across all 21 tests jointly. The likelihood ratio tests of model fit and of the equal-weight constraint, the tests of proportional hazards and log-linearity, and the subgroup, calendar-era, component, troponin, time-varying coefficient and other sensitivity analyses were not included in the adjustment and are reported with unadjusted *p* values; all analyses beyond the primary adjusted association are designated as exploratory. Full results are given in the Supplementary Material.

All tests were two-sided, and *p* < 0.05 was considered statistically significant. Analyses were performed with SPSS version 30.0 (IBM Corp., Armonk, NY, USA) and Python 3.13.5 (Python Software Foundation, Wilmington, DE, USA).

## 3. Results

### 3.1. Baseline Characteristics

Among the 2244 patients, MACEs occurred in 170 (7.6%) within one year, over a median follow-up of 400 days [interquartile range 366–400]. The components comprised 72 deaths, 42 myocardial infarctions, 25 ischemic strokes and 73 revascularizations. Baseline characteristics according to the occurrence of MACEs are shown in Table 1. Patients who experienced MACEs were older and had a greater burden of cardiovascular risk factors, renal dysfunction, anemia, impaired LVEF, multivessel disease and systemic inflammation. All four inflammatory indices were higher in the MACE group, whereas platelet count did not differ significantly between groups.

### 3.2. Association of the Indices with 1-Year MACE

In univariable analysis, all four log-transformed indices were strongly associated with MACEs, with hazard ratios between 1.49 and 1.54 per 1 SD increase (Table 2). After adjustment, NLR, SII and PIV remained independently associated with MACEs, whereas the association of SIRI was attenuated and did not reach significance: the adjusted HR per 1 SD was 1.21 (95% CI 1.04–1.42) for log(NLR), 1.17 (0.99–1.38) for log(SIRI), 1.26 (1.07–1.47) for log(SII) and 1.20 (1.03–1.41) for log(PIV). The proportional hazards assumption was satisfied for all four indices (*p* = 0.44 to 0.96). Restricted cubic spline analysis showed no significant departure from log-linearity for any of the four indices (*p* = 0.131 for SII, 0.342 for NLR, 0.491 for SIRI and 0.297 for PIV), so the associations are adequately summarized by the per 1 SD estimates.

### 3.3. Incremental Performance and Comparison Between Indices

The clinical model achieved a C-index of 0.682 and a 1-year AUC of 0.685 (Table 3 (A)). The addition of NLR, SII or PIV significantly improved model fit by the likelihood ratio test, whereas the addition of SIRI did not. The SII-added model showed the highest C-index (0.691), the highest AUC (0.698), the lowest AIC (2337.9) and the highest optimism-corrected C-index (0.672). When the increase in C-index was tested by paired bootstrap with 1000 resamples, however, no index significantly improved discrimination over the clinical model: ΔC-index +0.0094 (95% CI −0.0001 to +0.0246, *p* = 0.060) for SII, and *p* = 0.110, 0.110 and 0.178 for NLR, PIV and SIRI, respectively. In head-to-head bootstrap tests (Table 3 (B)), no statistically detectable difference was observed between the SII-added model and the NLR-added model (ΔC-index +0.0018, 95% CI −0.0024 to +0.0087, *p* = 0.382), the SIRI-added model (*p* = 0.298) or the PIV-added model (*p* = 0.404). The four indices are, therefore, best regarded as showing similar discriminative ability, without evidence that any one is superior.

### 3.4. Contribution of Platelet Count

Over the whole follow-up period, entering log-transformed platelet count as a free parameter alongside a platelet-free index did not significantly improve model fit (Table 3 (C)): the single-degree-of-freedom LRT gave *p* = 0.104 for the NLR-based model, in which platelet count carried an HR of 1.13 (95% CI 0.97–1.31) per 1 SD, and *p* = 0.146 for the SIRI-based model (HR 1.11, 95% CI 0.96–1.29). There was likewise no evidence against the equal-weight constraint imposed by the SII and PIV formulae (*p* = 0.563 and *p* = 0.449 against the corresponding unconstrained models). The more parsimonious SII model was slightly favored by AIC (2337.9 versus 2339.6 for the unconstrained model containing both log(NLR) and log(platelet)), although the difference was small.

### 3.5. Thirty-Day Landmark Analysis

After the exclusion of 51 patients with MACEs on or before day 30 and 18 patients lost to follow-up before day 30, the landmark cohort comprised 2175 patients, among whom 119 MACEs occurred between the landmark and one year. In this period, the two platelet-containing indices remained independently associated with MACEs—log(SII) HR 1.23 (95% CI 1.02–1.49, *p* = 0.030) and log(PIV) HR 1.22 (95% CI 1.01–1.48, *p* = 0.042)—whereas the two platelet-free indices did not (log(NLR) *p* = 0.169; log(SIRI) *p* = 0.163) (Table 4 (A)).

Repeating the decomposition within the landmark period yielded significant platelet terms, unlike the full-period analysis (Table 4 (B)). Adding log-transformed platelet count to the NLR-based model significantly improved fit (LRT *p* = 0.025), with platelet count associated with MACE occurring after the first 30 days (HR 1.22, 95% CI 1.03–1.45 per 1-SD); the corresponding test for the SIRI-based model was also significant (*p* = 0.033; HR 1.21, 95% CI 1.01–1.44).

The difference between periods was then tested directly. In the episode-split model over the whole follow-up (44 events on or before and 115 events after day 30), the estimated effect of log-transformed platelet count was HR 0.98 per 1 SD during the first 30 days and HR 1.20 thereafter, but the interaction between platelet count and period was not significant (ratio of hazard ratios 1.23, *p* = 0.197; *p* = 0.167 for the SIRI-based model). The corresponding interaction for log(NLR) was of borderline significance (*p* = 0.061), with the association being stronger in the first 30 days.

### 3.6. Cumulative Event Curves

**Figure 2:**
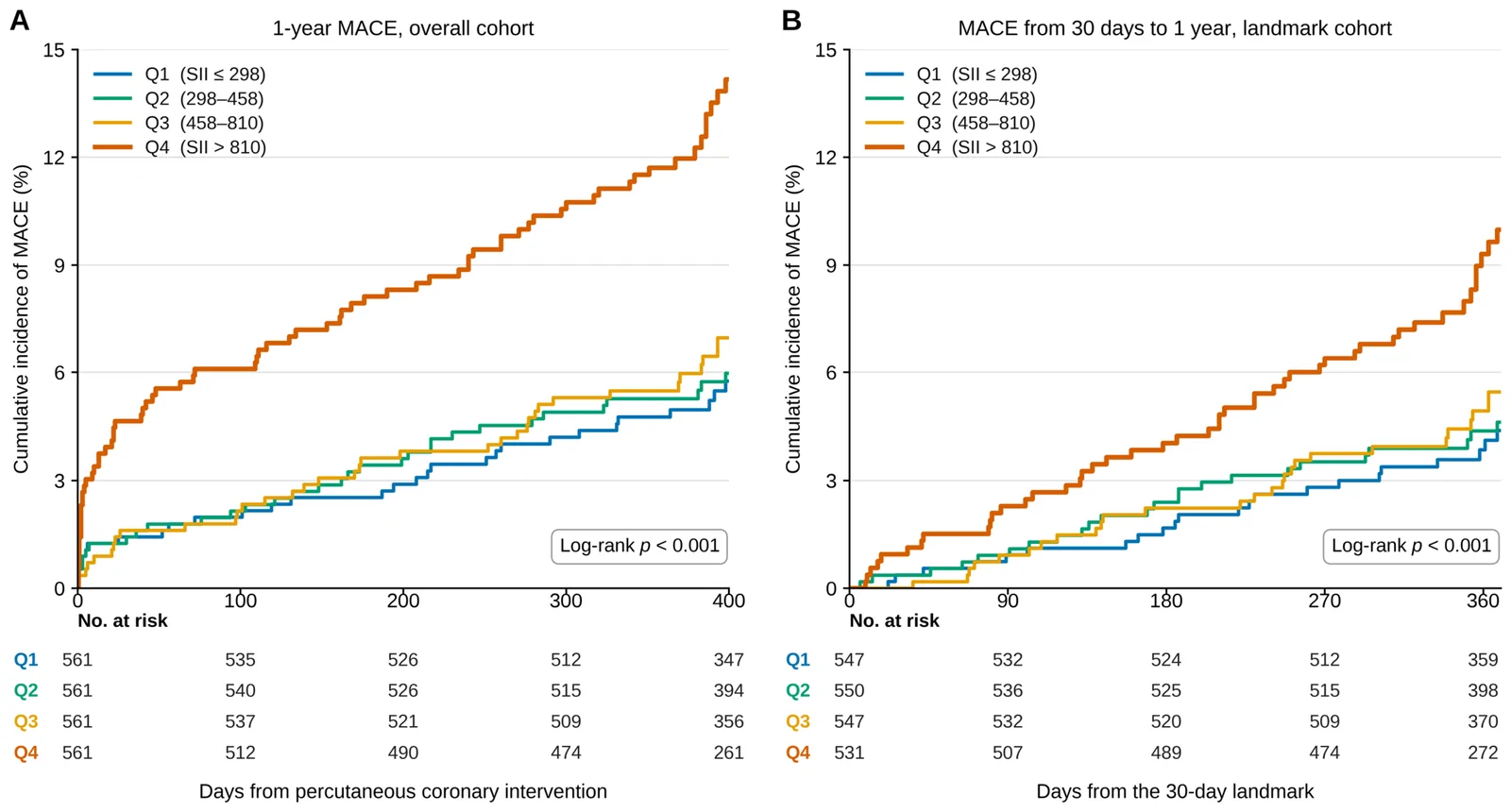
Cumulative incidence of major adverse cardiovascular events (MACEs) according to quartiles of the systemic immune–inflammation index (SII). (**A**) One-year MACEs in the overall cohort (*n* = 2244). (**B**) MACEs occurring between 30 days and one year in the 30-day landmark cohort (*n* = 2175). Curves are Kaplan–Meier estimates expressed as one minus the survival probability. Quartile cut points, derived from the overall cohort and applied to both panels, were 298, 458 and 810; values equal to a cut point were assigned to the lower quartile. Quartiles are used for description only.

Cumulative event curves according to quartiles of SII are shown in Figure 2. In the overall cohort, crude 1-year MACE proportions were 5.3%, 5.7%, 6.4% and 12.8% in the first through fourth quartiles (log-rank *p* < 0.001); in the landmark cohort, the corresponding crude MACE proportions from day 30 through day 400 were 4.0%, 4.4%, 4.9% and 8.7% (log-rank *p* < 0.001). Quartiles are used here for description only, and no cut-off was derived from these data.

### 3.7. Calibration and Clinical Utility

**Figure 3:**
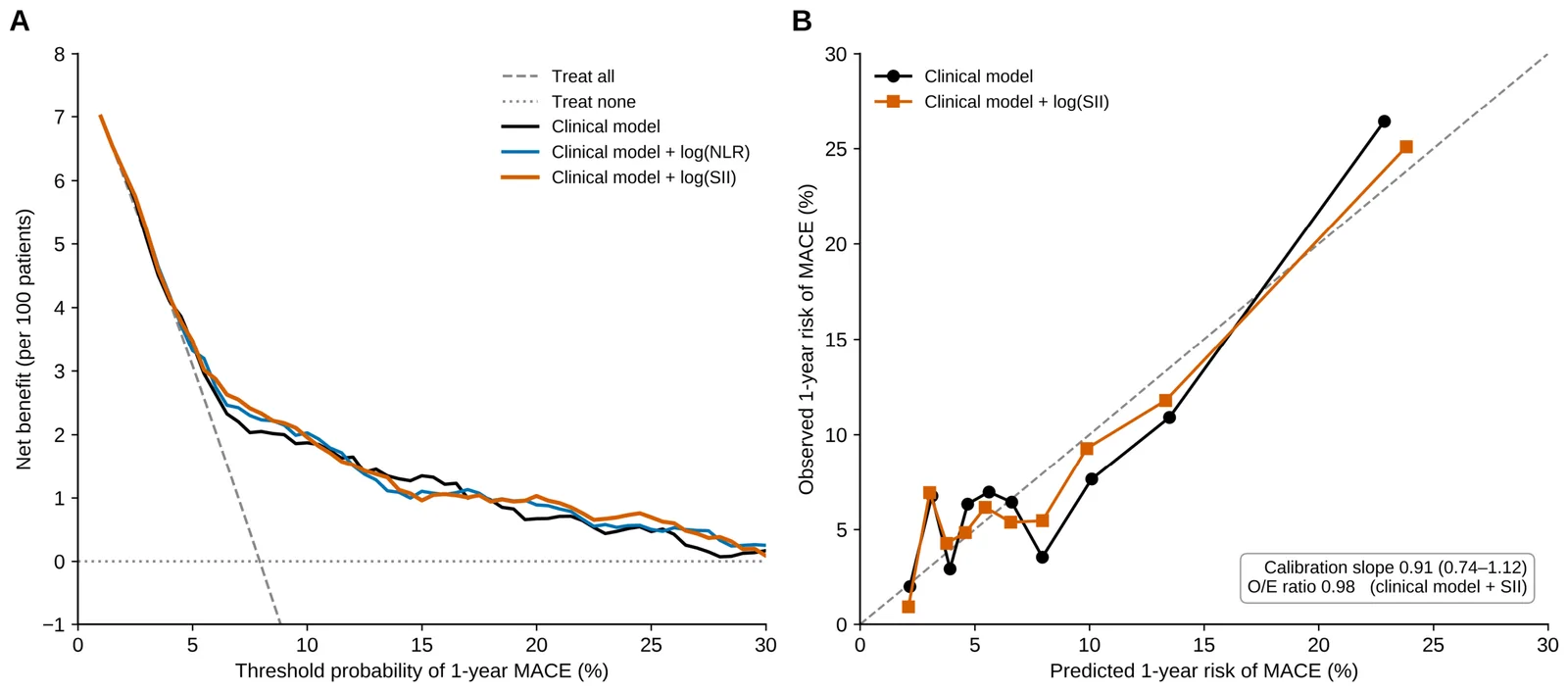
Clinical utility and calibration of the clinical model with and without inflammatory indices. (**A**) Decision curves for 1-year MACE, showing net benefit per 100 patients across threshold probabilities for the clinical model alone and with NLR or SII added, compared with treating all or no patients. (**B**) Calibration of the clinical model and the clinical model with SII: observed Kaplan–Meier 1-year risk plotted against mean predicted risk within deciles of predicted risk; the dashed line is the line of identity. MACEs, major adverse cardiovascular events; NLR, neutrophil-to-lymphocyte ratio; SII, systemic immune–inflammation index.

All models were well calibrated: the optimism-corrected calibration slope was 0.91 (95% CI 0.74–1.12) for the clinical model with SII, with an observed-to-expected ratio of 0.98 and close agreement between predicted and observed risk across deciles (Figure 3B; Table S7). Overall accuracy improved only marginally with any index: the Brier score at day 399 fell from 0.0689 for the clinical model to 0.0687 with SII, and the index of prediction accuracy rose from 5.7% to 6.0%. Decision-curve analysis (Figure 3A) showed that the clinical model provided net benefit over treating all or none across threshold probabilities of approximately 5% to 30%, but that adding any index shifted the curve only slightly: at a threshold of 7.5%, net benefit was 2.03 per 100 patients for the clinical model and 2.41 with SII, a difference of 0.38 net true-positive classifications per 100 patients; at 10%, the difference was +0.09.

### 3.8. Subgroup Analyses by Clinical Presentation

There was no evidence that the associations differed between acute and chronic coronary syndromes (*p* for interaction 0.22–0.51) and only weak evidence of a difference between MI and non-MI presentations (*p* 0.08–0.11), with point estimates consistently larger in MI (Table S1). When MI was divided into ST-segment elevation and non-ST-segment elevation MI, however, the associations were concentrated in ST-segment elevation MI: the univariable HR per 1 SD was 2.00 (95% CI 1.43–2.80) for SII and 1.94 (1.37–2.76) for NLR in that stratum, compared with 1.15 and 1.12 in non-ST-segment elevation MI and 1.13 and 1.07 in non-MI presentations, with significant index-by-presentation interaction for SII (*p* = 0.017) and NLR (*p* = 0.024) but not for SIRI or PIV. The ST-segment elevation stratum contained only 23 events (7 within 30 days and 16 thereafter; Table S1), so adjusted estimates could not be obtained within it, and this observation is exploratory. Calendar era had no influence: estimates were unchanged after adjustment or stratification by era, era was not itself associated with MACEs, and no index-by-era interaction was present (*p* 0.30–0.56; Table S2).

### 3.9. Sensitivity Analyses

Censoring at 365 days (150 events) left the adjusted association of SII unchanged (HR 1.23, 95% CI 1.04–1.45, *p* = 0.015); of the 20 first events accrued between day 366 and day 400, 11 were revascularizations, six were deaths and three were strokes (Table S3). In component analyses, all four indices were associated univariably with all-cause death and ischemic stroke but not with MI or revascularization; none of the adjusted associations with death reached statistical significance, although the estimates for NLR and SII were borderline (Table S4). Adjustment for admission or peak troponin T, expressed as within-assay-era normal scores, did not materially alter the index estimates (SII HR 1.26 to 1.24). In the model containing SII, neither admission nor peak troponin T was independently associated with MACEs, and platelet count showed essentially no correlation with troponin T (Spearman |ρ| ≤ 0.05), so the platelet decomposition was unaffected (Table S5). Only 81 patients (3.6%) had a missing covariate, and multiple imputation reproduced the complete-case estimates (Table S6).

Time-varying coefficient models (Table S8; Figure S1) placed the point estimates for platelet count in the intermediate period rather than uniformly after day 30: the adjusted HR per 1 SD was 0.97 in days 0–30, 1.66 in days 31–90, 1.35 in days 91–180 and 1.06 in days 181–400, but heterogeneity across periods was not significant (*p* = 0.137) and there was no significant linear trend in log(time) (*p* = 0.109 by the Schoenfeld score test; *p* = 0.243 in the event-time-split model). For NLR, the point estimate was larger in the first 30 days (HR 1.48) than thereafter (HR 1.09–1.21), but again neither heterogeneity (*p* = 0.305) nor a trend in log(time) (*p* = 0.989 and 0.247) was significant. After within-family Holm correction, only the adjusted association between SII and MACEs remained significant (adjusted *p* = 0.016). When all 21 tests, including the primary analysis, were pooled in a single Benjamini–Hochberg analysis, no test met the 5% false-discovery-rate threshold (Table S9). Excluding the 12 patients with discordant event records described in Section 2.4 changed the adjusted HR for SII from 1.26 (95% CI 1.07–1.47) to 1.27 (1.08–1.48).

## 4. Discussion

In this all-comer PCI cohort, three findings are central. First, SII had the most favorable point estimates for discrimination and model fit, but no index significantly improved discrimination over the clinical model, no head-to-head difference was detected, and gains in net benefit were negligible. Second, adding platelet count did not significantly improve model fit over the full follow-up; a post-30-day signal was not supported by formal interaction or four-period heterogeneity testing. Third, larger associations observed in STEMI were exploratory and based on few events. After within-family Holm correction, only the adjusted SII–MACE association remained significant, and no test met the 5% false-discovery-rate threshold when all tests were pooled; every other result should be regarded as exploratory.

Previous cohort studies and meta-analyses have established associations of SII and PIV with post-PCI outcomes, particularly in STEMI, but formal head-to-head comparisons of incremental performance remain limited [5,7,8,17,18,19]. In our cohort, the difference in C-index between the SII- and NLR-added models was only 0.0018, with a confidence interval spanning zero. Given the limited number of events, these findings do not establish equivalence, but they do not support a clinically important advantage of SII. More importantly, the best-performing index increased the C-index by only 0.009, the IPA by approximately 0.4 percentage points and net benefit by fewer than one classification per 100 patients. Thus, the indices were associated with outcome but added little clinical utility beyond the clinical model, and these results should be read as a comparative study of model performance rather than as support for a new clinically actionable biomarker.

Because SII and PIV equal their platelet-free counterparts multiplied by platelet count, nested models allowed the platelet component to be tested directly. Platelet count did not significantly improve model fit over the full follow-up, and there was no evidence against the equal weighting that the formulae impose. Although a signal emerged after the 30-day landmark, neither the platelet-by-period interaction nor the four-period heterogeneity test was significant, and the landmark findings did not survive multiplicity correction. The data, therefore, do not establish temporal effect modification, and the post-30-day signal should be regarded as hypothesis-generating.

A time-restricted contribution of platelet count would be biologically plausible. Early post-PCI events may be more strongly influenced by infarct severity, hemodynamic compromise, procedural injury and acute inflammation, which may be reflected in leukocyte-derived indices and hsCRP. Baseline platelet count may capture aspects of the underlying thrombo-inflammatory milieu that become more discernible once the dominant influence of early ischemic and procedural risk has subsided [1,20,21]. This interpretation remains hypothetical for two reasons. Platelet count is not a direct measure of platelet activation or of platelet–leukocyte interactions, which also operate in acute coronary syndromes and in acute thrombus formation rather than only over longer time scales; in addition, more generally, blood-count-derived indices are distant surrogates of the inflammatory and resolution processes in adipose, vascular and myocardial tissue that drive cardiovascular disease, so their behavior should not be equated with that of the underlying biology [22]. The absence of correlation with troponin T and the stability of the estimates after troponin adjustment argue against, but do not exclude, confounding by infarct size.

The concentration of the associations in ST-segment elevation MI is consistent with the literature, in which the strongest and most consistent reports of these indices come from that population [7,17,18,19], and with the distinct inflammatory and thrombotic profile of this presentation described in current guidance [23]. It also cautions against pooling presentations: adjustment for a single MI-presentation term evidently did not capture this heterogeneity, and although the interaction was significant only for SII and NLR, the ST-segment elevation stratum contained too few events for adjusted estimation. Whether the indices carry more information in ST-segment elevation MI, or whether they simply track the acute leukocyte response to a large infarct, cannot be resolved here.

These indices are part of a broader group of inexpensive biomarkers available in admission blood samples. Stress hyperglycemia measures have also been associated with periprocedural myocardial injury and subsequent events after MI [24,25], underscoring the links among inflammatory, metabolic and thrombotic pathways. Whether combinations of these markers improve prediction should be evaluated using calibration and decision-curve analysis rather than association measures alone. Time-stratified analyses may be informative, but the temporal platelet pattern observed here requires independent confirmation.

This study has several limitations. It was a retrospective analysis from a single tertiary center, and residual confounding cannot be excluded; in particular, Killip class was recorded in fewer than half of the patients and could not be included, and the interval between symptom onset and blood sampling was not available, although sampling preceded angiography and all acute pharmacological treatment. The indices were derived from a single baseline blood count, and serial measurements were unavailable. Platelet count does not measure platelet activation, and total monocyte count does not distinguish monocyte subsets. The number of events limits the power of the head-to-head comparisons, of the interaction tests, and of the presentation subgroups, and the STEMI stratum in particular was too small for adjusted estimation. The analyses generate many tests, and although we have applied multiplicity adjustment and designated all secondary results as exploratory, the landmark analysis was planned before analysis rather than pre-registered. Follow-up was administratively censored at 400 days, so these findings do not extend to longer-term risk. Finally, the models were internally validated by bootstrap optimism correction only; the cohort was recruited over a decade at one center, and validation in an independent PCI population would be required before any broader applicability could be claimed.

## 5. Conclusions

Among complete blood count-derived inflammatory indices, SII had the most favorable point estimates for discrimination and model fit, but no index significantly improved discrimination beyond the clinical model, no head-to-head difference was detected, and gains in net benefit were negligible. Platelet count did not improve model fit over the full follow-up; a signal observed after day 30 was not supported by formal interaction or time-varying analyses and remains hypothesis-generating. Exploratory estimates were largest in STEMI but were based on few events. After within-family Holm correction, only the adjusted SII–MACE association remained significant, whereas no result met the 5% false-discovery-rate threshold in the global analysis.

## Figures and Tables

**Table 1:** Baseline characteristics according to the occurrence of major adverse cardiovascular events within one year.

Variable	MACEs (*n* = 170)	No MACEs (*n* = 2074)	*p* Value
***Demographics and risk factors***			
Age, years	70.2 ± 10.2	65.4 ± 11.3	<0.001
Male sex	111 (65.3)	1437 (69.3)	0.319
Hypertension	131 (77.1)	1295 (62.4)	<0.001
Diabetes mellitus	88 (51.8)	810 (39.1)	0.002
Current smoking	42 (24.7)	648 (31.2)	0.091
Previous myocardial infarction	9 (5.3)	120 (5.8)	0.926
Previous PCI	17 (10.0)	194 (9.4)	0.888
Previous CABG	3 (1.8)	20 (1.0)	0.548
Previous stroke	30 (17.6)	201 (9.7)	0.002
***Clinical presentation***			
Clinical diagnosis			<0.001
SAP	25 (14.7)	430 (20.7)	
UAP	48 (28.2)	769 (37.1)	
NSTEMI	74 (43.5)	496 (23.9)	
STEMI	23 (13.5)	379 (18.3)	
Left ventricular ejection fraction, %	51.5 ± 14.9	55.8 ± 12.2	<0.001
***Laboratory findings***			
White blood cell, 10^3^/μL	8.63 [6.63–11.92]	7.94 [6.41–10.19]	0.009
Neutrophil, 10^3^/μL	5.82 [3.63–8.99]	4.59 [3.45–6.68]	<0.001
Lymphocyte, 10^3^/μL	1.95 [1.38–2.48]	2.21 [1.66–2.84]	<0.001
Monocyte, 10^3^/μL	0.56 [0.45–0.75]	0.53 [0.42–0.70]	0.071
Platelet, 10^3^/μL	234.1 ± 69.1	229.3 ± 63.3	0.379
Hemoglobin, g/dL	12.7 ± 2.3	13.6 ± 2.0	<0.001
eGFR, mL/min/1.73 m^2^	74.5 [50.1–91.4]	86.2 [68.3–98.0]	<0.001
LDL-cholesterol, mg/dL	92.5 [69.0–121.2]	105.0 [78.0–132.0]	0.006
hsCRP, mg/L	2.33 [0.91–7.58]	1.44 [0.73–3.77]	<0.001
***Systemic inflammatory indices***			
NLR	2.86 [1.65–5.69]	2.02 [1.39–3.36]	<0.001
SIRI	1.55 [0.82–3.71]	1.08 [0.67–1.96]	<0.001
SII	618 [338–1443]	447 [295–769]	<0.001
PIV	326 [177–944]	237 [140–454]	<0.001
***Angiographic and procedural characteristics***			
Multivessel disease	106 (62.4)	912 (44.0)	<0.001
Complex PCI	52 (30.6)	498 (24.0)	0.068
Chronic total occlusion	11 (6.5)	116 (5.6)	0.762
Bifurcation with two stents	4 (2.4)	38 (1.8)	0.851
Intravascular ultrasound guidance	28 (16.5)	358 (17.3)	0.875
Number of treated lesions	1 [1,2]	1 [1,2]	0.866
Number of stents	1 [1,2]	1 [1,2]	0.274
Minimal stent diameter, mm	2.8 [2.5–3.0]	3.0 [2.5–3.0]	0.014
Total stent length, mm	36 [22–62]	33 [23–54]	0.182

Values are mean ± standard deviation, median [interquartile range] or n (%). *p* values were derived from the Welch *t*-test, the Mann–Whitney *U* test or the chi-square test. CABG, coronary artery bypass grafting; eGFR, estimated glomerular filtration rate; hsCRP, high-sensitivity C-reactive protein; LDL, low-density lipoprotein; MACEs, major adverse cardiovascular events; NLR, neutrophil-to-lymphocyte ratio; NSTEMI, non-ST-segment elevation myocardial infarction; PCI, percutaneous coronary intervention; PIV, pan-immune–inflammation value; SAP, stable angina pectoris; SII, systemic immune–inflammation index; SIRI, systemic inflammatory response index; STEMI, ST-segment elevation myocardial infarction; UAP, unstable angina pectoris.

**Table 2:** Cox regression analysis for 1-year major adverse cardiovascular events according to systemic inflammatory indices.

Variable	Univariable HR (95% CI)	*p* Value	Adjusted HR (95% CI)	*p* Value
log(NLR)	1.54 (1.35–1.76)	<0.001	1.21 (1.04–1.42)	0.017
log(SIRI)	1.49 (1.31–1.71)	<0.001	1.17 (0.99–1.38)	0.059
log(SII)	1.54 (1.34–1.76)	<0.001	1.26 (1.07–1.47)	0.004
log(PIV)	1.49 (1.30–1.71)	<0.001	1.20 (1.03–1.41)	0.024

Hazard ratios are expressed per 1-standard-deviation increase in the natural log-transformed index. The adjusted model included age, sex, hypertension, diabetes mellitus, myocardial infarction presentation, hemoglobin, eGFR, log-transformed hsCRP, multivessel disease and left ventricular ejection fraction (*n* = 2163; 159 events). The univariable analysis included all 2244 patients (170 events). CI, confidence interval; HR, hazard ratio.

**Table 3:** Incremental predictive performance of systemic inflammatory indices for 1-year major adverse cardiovascular events, and the contribution of platelet count. (**A**) Incremental value of each index beyond the clinical model. (**B**) Head-to-head comparison of discrimination. (**C**) Decomposition of the platelet contribution (nested 1-degree-of-freedom tests).

(**A**)									
**Model**	**C-Index**	**ΔC-Index (95% CI)**		***p* Value**	**1-Year AUC**	**AIC**	**ΔAIC**	**LRT *p* Value**	**Optimism-Corrected C-Index**
Clinical model	0.682	Reference		Ref.	0.685	2343.8	Ref.	Reference	0.663
+log(NLR)	0.689	+0.0076 (−0.0004 to +0.0200)		0.110	0.695	2340.2	−3.6	0.018	0.669
+log(SIRI)	0.687	+0.0048 (−0.0008 to +0.0165)		0.178	0.692	2342.3	−1.5	0.061	0.668
+log(SII)	0.691	+0.0094 (−0.0001 to +0.0246)		0.060	0.698	2337.9	−5.9	0.005	0.672
+log(PIV)	0.688	+0.0066 (−0.0005 to +0.0201)		0.110	0.694	2340.8	−3.0	0.025	0.668
(**B**)									
**Comparison**				**Difference in C-Index**				**95% CI**	***p* Value**
Clinical model + log(SII) vs. clinical model + log(NLR)				+0.0018				−0.0024 to +0.0087	0.382
Clinical model + log(SII) vs. clinical model + log(SIRI)				+0.0046				−0.0024 to +0.0146	0.298
Clinical model + log(SII) vs. clinical model + log(PIV)				+0.0028				−0.0032 to +0.0116	0.404
(**C**)									
**Model**		**C-Index**	**AIC**	**LRT *p* Value for Adding log(platelet)**		**LRT *p* Value for the Equal-Weight Constraint**		**HR for log(platelet) per 1 SD (95% CI)**	
Clinical model + log(NLR)		0.689	2340.2	–		–		–	
+log(platelet)		0.692	2339.6	0.104		–		1.13 (0.97–1.31)	
Clinical model + log(SII)		0.691	2337.9	–		0.563		–	
Clinical model + log(SIRI)		0.687	2342.3	–		–		–	
+log(platelet)		0.689	2342.2	0.146		–		1.11 (0.96–1.29)	
Clinical model + log(PIV)		0.688	2340.8	–		0.449		–	

All models were fitted on the same complete-case sample (n = 2163; 159 events) so that AIC values and likelihood ratio tests are directly comparable. Lower AIC values indicate better model fit. The clinical model comprised age, sex, hypertension, diabetes mellitus, myocardial infarction presentation, hemoglobin, eGFR, log-transformed hsCRP, multivessel disease and left ventricular ejection fraction. The optimism-corrected C-index was obtained by bootstrap internal validation with 1000 resamples. The 1-year AUC is the inverse-probability-of-censoring-weighted cumulative/dynamic AUC evaluated at day 399, the last evaluable time point. Differences in C-index were tested by paired bootstrap with 1000 resamples. In Panel C, “+ log(platelet)” denotes addition of standardized log-transformed platelet count as a free parameter to the model in the preceding row; the equal-weight constraint test compares that unconstrained model with the corresponding platelet-containing index model, which, on the logarithmic scale, constrains the coefficients of the two components to be equal. AIC, Akaike information criterion; AUC, area under the curve; LRT, likelihood ratio test.

**Table 4:** Thirty-day landmark analysis for major adverse cardiovascular events occurring between 30 days and one year after percutaneous coronary intervention. (**A**) Per 1 SD increase in log-transformed index, (**B**) decomposition of the platelet contribution within the landmark period.

(**A**)					
**Variable**	**Univariable HR (95% CI)**		***p* Value**	**Adjusted HR (95% CI)**	***p* Value**
log(NLR)	1.41 (1.20–1.66)		<0.001	1.15 (0.94–1.39)	0.169
log(SIRI)	1.42 (1.21–1.68)		<0.001	1.15 (0.94–1.40)	0.163
log(SII)	1.44 (1.22–1.70)		<0.001	1.23 (1.02–1.49)	0.030
log(PIV)	1.44 (1.22–1.70)		<0.001	1.22 (1.01–1.48)	0.042
(**B**)					
**Model**	**C-Index**	**AIC**	**LRT *p* Value for Adding log(platelet)**	**LRT *p* Value for the Equal-Weight Constraint**	**HR for log(platelet) per 1 SD (95% CI)**
Clinical model + log(NLR)	0.674	1703.1	–	–	–
+ log(platelet)	0.681	1700.0	0.025	–	1.22 (1.03–1.45)
Clinical model + log(SII)	0.679	1700.4	–	0.128	–
Clinical model + log(SIRI)	0.674	1703.0	–	–	–
+ log(platelet)	0.681	1700.5	0.033	–	1.21 (1.01–1.44)
Clinical model + log(PIV)	0.678	1700.9	–	0.123	–

The landmark cohort comprised 2175 patients after exclusion of 51 patients with MACEs on or before day 30 and 18 patients lost to follow-up before day 30, and 119 MACEs occurred between day 30 and day 400. Follow-up time was measured from the landmark. The univariable analysis included all 2175 patients, and the adjusted analysis, which used the same covariates as those in Table 2, included 2101 patients (115 events); Panel B was fitted on the same complete-case sample. Abbreviations as in Table 2 and Table 3.

## Data Availability

The datasets generated and analyzed during the current study are not publicly available because of privacy and ethical restrictions, but are available from the corresponding author on reasonable request and with the permission of the Institutional Review Board of Wonkwang University Hospital.

## Supplementary Materials

The following supporting information can be downloaded at: https://www.mdpi.com/article/10.3390/jcm15176872/s1, Table S1. Association of each index with 1-year major adverse cardiovascular events according to clinical presentation, with tests of interaction. Table S2. Sensitivity of the adjusted associations to calendar era of the index procedure. Table S3. Sensitivity analysis with follow-up censored at 365 days. Table S4. Association of each index with the individual components of major adverse cardiovascular events within 400 days. Table S5. Sensitivity analysis adjusting for troponin T (within-assay-era normal scores). Table S6. Missing covariate data and multiple-imputation sensitivity analysis. Table S7. Calibration, overall accuracy and clinical utility of the clinical model with and without each index. Table S8. Period-specific (piecewise-constant) hazard ratios for log(platelet) and log(NLR). Table S9. Multiplicity-adjusted *p* values for the principal inferential tests. Figure S1. Time-varying effect of platelet count and NLR on 1-year MACE.

## Abbreviations

The following abbreviations are used in this manuscript:

AIC	Akaike information criterion
AUC	area under the receiver operating characteristic curve
CI	confidence interval
eGFR	estimated glomerular filtration rate
HR	hazard ratio
hsCRP	high-sensitivity C-reactive protein
LRT	likelihood ratio test
LVEF	left ventricular ejection fraction
MACE	major adverse cardiovascular events
MI	myocardial infarction
NLR	neutrophil-to-lymphocyte ratio
PCI	percutaneous coronary intervention
PIV	pan-immune–inflammation value
SD	standard deviation
SII	systemic immune–inflammation index
SIRI	systemic inflammatory response index
