# Supplementary material for: Comparative Prognostic Performance of Platelet-Containing and Platelet-Free Inflammatory Indices After Percutaneous Coronary Intervention
Source: J Clin Med. 2026 Sep 4;15(17):6872. doi: 10.3390/jcm15176872 (PMC13566493; doi:10.3390/jcm15176872)
Supplement: Supplementary file 1 [file jcm-15-06872-s001.zip › jcm-4520432-supplementary.pdf]

Supplementary Material

Comparative Prognostic Performance of Platelet-Containing and Platelet-Free Inflammatory Indices After Percutaneous Coronary Intervention

Donghyeon Joo, Sungho Jo, Jeong Tae Byoun, Jae Young Cho and Kyeong Ho Yun

Contents

Table S1. Association of each index with 1-year major adverse cardiovascular events according to clinical presentation, with tests of interaction

Table S2. Sensitivity of the adjusted associations to calendar era of the index procedure

Table S3. Sensitivity analysis with follow-up censored at 365 days

Table S4. Association of each index with the individual components of major adverse cardiovascular events within 400 days

Table S5. Sensitivity analysis adjusting for troponin T (within-assay-era normal scores)

Table S6. Missing covariate data and multiple-imputation sensitivity analysis

Table S7. Calibration, overall accuracy and clinical utility of the clinical model with and without each index

Table S8. Period-specific (piecewise-constant) hazard ratios for log(platelet) and log(NLR)

Table S9. Multiplicity-adjusted p values for the principal inferential tests

Figure S1. Time-varying effect of platelet count and NLR on 1-year MACE

Table S1. Association of each index with 1-year major adverse cardiovascular events according to clinical presentation, with tests of interaction.

Panel A. Acute coronary syndrome versus chronic coronary syndrome

Index	ACS	HR (95% CI)	p	CCS	HR (95% CI)	p	p for interaction
log(NLR)	(n = 1727, 136 events)	1.24 (1.05–1.46)	0.012	(n = 455, 25 events)	1.52 (0.99–2.34) †	0.054	0.244
log(SIRI)	(n = 1727, 136 events)	1.17 (0.99–1.39)	0.071	(n = 455, 25 events)	1.52 (1.01–2.31) †	0.046	0.512
log(SII)	(n = 1727, 136 events)	1.28 (1.09–1.51)	0.003	(n = 455, 25 events)	1.41 (0.91–2.18) †	0.124	0.220
log(PIV)	(n = 1727, 136 events)	1.21 (1.02–1.43)	0.027	(n = 455, 25 events)	1.44 (0.94–2.21) †	0.097	0.469

Panel B. Myocardial infarction versus no myocardial infarction

Index	MI	HR (95% CI)	p	Non-MI	HR (95% CI)	p	p for interaction
log(NLR)	(n = 945, 91 events)	1.25 (1.04–1.51)	0.018	(n = 1218, 68 events)	1.07 (0.76–1.50)	0.704	0.090
log(SIRI)	(n = 945, 91 events)	1.19 (0.99–1.45)	0.067	(n = 1218, 68 events)	1.01 (0.73–1.41)	0.932	0.105
log(SII)	(n = 945, 91 events)	1.29 (1.07–1.55)	0.008	(n = 1218, 68 events)	1.13 (0.83–1.56)	0.438	0.084
log(PIV)	(n = 945, 91 events)	1.22 (1.01–1.47)	0.039	(n = 1218, 68 events)	1.07 (0.78–1.46)	0.667	0.106

Panel C. Non-MI versus NSTEMI versus STEMI

Index	Non-MI	HR (95% CI)	p	NSTEMI	HR (95% CI)	p	STEMI	HR (95% CI)	p	p for interaction
log(NLR)	(68/1218)	1.07 (0.76–1.50)	0.704	(68/547)	1.12 (0.90–1.40)	0.300	(23/402)	1.94 (1.37–2.76) †	<0.001	0.024
log(SIRI)	(68/1218)	1.01 (0.73–1.41)	0.932	(68/547)	1.12 (0.90–1.40)	0.292	(23/402)	1.81 (1.23–2.68) †	0.003	0.104
log(SII)	(68/1218)	1.13 (0.83–1.56)	0.438	(68/547)	1.15 (0.92–1.43)	0.213	(23/402)	2.00 (1.43–2.80) †	<0.001	0.017
log(PIV)	(68/1218)	1.07 (0.78–1.46)	0.667	(68/547)	1.14 (0.92–1.42)	0.229	(23/402)	1.88 (1.29–2.72) †	<0.001	0.082

Panel D. Platelet decomposition within the 30-day landmark period, by presentation

Presentation	Model	n / events	HR for log(platelet) per 1 SD (95% CI)	LRT p (1 df)
MI	NLR-based	904 / 59	1.30 (1.03–1.64)	0.026
MI	SIRI-based	904 / 59	1.28 (1.01–1.62)	0.036
Non-MI	NLR-based	1197 / 56	1.10 (0.84–1.44)	0.475
Non-MI	SIRI-based	1197 / 56	1.10 (0.84–1.44)	0.482

The adjusted model included age, sex, hypertension, diabetes mellitus, myocardial infarction presentation, hemoglobin, eGFR, log-transformed hsCRP, multivessel disease and left ventricular ejection fraction. Hazard ratios are expressed per 1-standard-deviation increase in the natural log-transformed index. In the MI, non-MI, NSTEMI and STEMI strata, MI presentation was omitted from the adjustment set because it is constant within the stratum. Subgroup columns give n and the number of events (Panels A and B) or events/n (Panel C).

† Univariable estimate. Strata with fewer than five events per model parameter (CCS 25 events; STEMI 23 events; 10 parameters) were not adjusted. Interaction p values are from likelihood ratio tests of index-by-presentation product terms in the fully adjusted model fitted to the whole cohort. The MI-presentation term of the adjusted model entered these tests as follows: in Panel A it was retained, and the ACS indicator and its product with the index were added (the two terms jointly encode a three-level main effect of chronic coronary syndrome, unstable angina and MI; replacing the MI term by the ACS indicator instead gave p = 0.151 for SII); in Panel B it is itself the grouping variable, and only the product term was added; in Panel C it was replaced by indicators for NSTEMI and STEMI, which jointly encode MI presentation, and two product terms were added (2 degrees of freedom). In the ACS stratum the MI term was retained because presentation varies within ACS. Of the 23 MACE in the STEMI stratum, 7 occurred within 30 days and 16 thereafter. Crude 1-year MACE proportions were 25/455 (5.5%) for stable angina, 48/817 (5.9%) for unstable angina, 74/570 (13.0%) for NSTEMI and 23/402 (5.7%) for STEMI. Given the number of tests and the small number of STEMI events, the interaction observed for NLR and SII in Panel C should be regarded as exploratory.

ACS, acute coronary syndrome; CCS, chronic coronary syndrome; CI, confidence interval; eGFR, estimated glomerular filtration rate; HR, hazard ratio; hsCRP, high-sensitivity C-reactive protein; LRT, likelihood ratio test; MACE, major adverse cardiovascular events; NLR, neutrophil-to-lymphocyte ratio; NSTEMI, non-ST-segment elevation myocardial infarction; PIV, pan-immune-inflammation value; SD, standard deviation; SII, systemic immune-inflammation index; SIRI, systemic inflammatory response index; STEMI, ST-segment elevation myocardial infarction.

Table S2. Sensitivity of the adjusted associations to calendar era of the index procedure.

Index	Main model HR (95% CI)	p	Era-adjusted HR (95% CI)	p	Era-stratified HR (95% CI)	p	p for index-by-era interaction
log(NLR)	1.21 (1.04–1.42)	0.017	1.22 (1.04–1.43)	0.016	1.21 (1.04–1.42)	0.017	0.296
log(SIRI)	1.17 (0.99–1.38)	0.059	1.17 (0.99–1.38)	0.064	1.17 (0.99–1.38)	0.064	0.344
log(SII)	1.26 (1.07–1.47)	0.004	1.26 (1.07–1.47)	0.005	1.26 (1.07–1.47)	0.005	0.530
log(PIV)	1.20 (1.03–1.41)	0.024	1.20 (1.02–1.41)	0.027	1.20 (1.02–1.41)	0.026	0.564

The adjusted model included age, sex, hypertension, diabetes mellitus, myocardial infarction presentation, hemoglobin, eGFR, log-transformed hsCRP, multivessel disease and left ventricular ejection fraction. Hazard ratios are expressed per 1-standard-deviation increase in the natural log-transformed index. Calendar era was defined as 2012–2016 (n = 1002; 66 MACE) versus 2017–2021 (n = 1242; 104 MACE) and was either added as a covariate (era-adjusted) or used as a stratification variable with era-specific baseline hazards (era-stratified). Era itself was not associated with MACE after adjustment (HR 1.12, 95% CI 0.82–1.55, $p = 0.47$).

CI, confidence interval; eGFR, estimated glomerular filtration rate; HR, hazard ratio; hsCRP, high-sensitivity C-reactive protein; MACE, major adverse cardiovascular events; NLR, neutrophil-to-lymphocyte ratio; PIV, pan-immune-inflammation value; SD, standard deviation; SII, systemic immune-inflammation index; SIRI, systemic inflammatory response index.

Table S3. Sensitivity analysis with follow-up censored at 365 days.

Panel A. Association and incremental value

Index	Univariable HR (95% CI)	p	Adjusted HR (95% CI)	p	C-index	AIC	ΔAIC	LRT p
log(NLR)	1.54 (1.33–1.77)	<0.001	1.20 (1.01–1.42)	0.036	0.693	2070.0	-2.3	0.038
log(SIRI)	1.49 (1.29–1.72)	<0.001	1.15 (0.97–1.37)	0.108	0.691	2071.8	-0.6	0.110
log(SII)	1.52 (1.32–1.75)	<0.001	1.23 (1.04–1.45)	0.015	0.695	2068.5	-3.8	0.016
log(PIV)	1.47 (1.27–1.70)	<0.001	1.18 (0.99–1.40)	0.059	0.692	2070.8	-1.5	0.060

Panel B. Platelet decomposition over the full period

Model	HR for log(platelet) per 1 SD (95% CI)	LRT p (1 df)
NLR-based	1.11 (0.95–1.29)	0.203
SIRI-based	1.09 (0.94–1.28)	0.261

MACE within 365 days occurred in 150 patients (univariable analysis); the adjusted analysis included 2163 patients with 140 events. The clinical model alone had a C-index of 0.689 and an AIC of 2072.3; ΔAIC and the LRT compare each index-added model with the clinical model. The adjusted model included age, sex, hypertension, diabetes mellitus, myocardial infarction presentation, hemoglobin, eGFR, log-transformed hsCRP, multivessel disease and left ventricular ejection fraction. Hazard ratios are expressed per 1-standard-deviation increase in the natural log-transformed index.

Of the 20 first events between day 366 and day 400 in the main analysis, 11 were revascularizations (target-vessel in 5, of which 3 were target-lesion; non-target-vessel in 6), 6 were deaths (one with a concurrent myocardial infarction) and 3 were ischemic strokes. Counts of patients with a first occurrence of each component by 365 versus 400 days were: death 66 versus 72, myocardial infarction 41 versus 42, ischemic stroke 22 versus 25 and any revascularization 62 versus 73. Clinical follow-up was scheduled at one year, and revascularization prompted by symptoms elicited at that visit is typically performed at a scheduled admission within the following weeks; the 400-day window was chosen to capture these procedures.

AIC, Akaike information criterion; CI, confidence interval; eGFR, estimated glomerular filtration rate; HR, hazard ratio; hsCRP, high-sensitivity C-reactive protein; LRT, likelihood ratio test; MACE, major adverse cardiovascular events; MI, myocardial infarction; NLR, neutrophil-to-lymphocyte ratio; PIV, pan-immune-inflammation value; SD, standard deviation; SII, systemic immune-inflammation index; SIRI, systemic inflammatory response index.

Table S4. Association of each index with the individual components of major adverse cardiovascular events within 400 days.

Component	Events	Index	Univariable HR (95% CI)	p	Adjusted HR (95% CI)	p
All-cause death	72	log(NLR)	1.92 (1.60–2.32)	<0.001	1.25 (0.98–1.58)	0.071
		log(SIRI)	1.84 (1.52–2.22)	<0.001	1.18 (0.93–1.51)	0.180
		log(SII)	1.85 (1.52–2.24)	<0.001	1.23 (0.97–1.57)	0.086
		log(PIV)	1.77 (1.45–2.16)	<0.001	1.17 (0.92–1.49)	0.204
Myocardial infarction	42	log(NLR)	1.13 (0.84–1.52)	0.427	– ‡	
		log(SIRI)	1.16 (0.87–1.56)	0.318	– ‡	
		log(SII)	1.16 (0.86–1.56)	0.327	– ‡	
		log(PIV)	1.18 (0.88–1.59)	0.259	– ‡	
Ischemic stroke	25	log(NLR)	1.49 (1.05–2.12)	0.024	– ‡	
		log(SIRI)	1.55 (1.10–2.19)	0.013	– ‡	
		log(SII)	1.56 (1.11–2.21)	0.011	– ‡	
		log(PIV)	1.60 (1.13–2.27)	0.008	– ‡	
Any revascularization	73	log(NLR)	1.17 (0.94–1.47)	0.165	1.14 (0.88–1.48)	0.314
		log(SIRI)	1.13 (0.90–1.42)	0.280	1.08 (0.83–1.41)	0.569
		log(SII)	1.21 (0.96–1.51)	0.102	1.21 (0.94–1.56)	0.140
		log(PIV)	1.16 (0.93–1.45)	0.197	1.14 (0.88–1.48)	0.326

Each component was analyzed as time to its first occurrence, censoring at day 400 or at the end of follow-up; components are not mutually exclusive. The adjusted model included age, sex, hypertension, diabetes mellitus, myocardial infarction presentation, hemoglobin, eGFR, log-transformed hsCRP, multivessel disease and left ventricular ejection fraction. Hazard ratios are expressed per 1-standard-deviation increase in the natural log-transformed index.

‡ Not adjusted: components with fewer than five events per model parameter (MI 42 events; stroke 25 events; 11 parameters) were analyzed univariably only, because the number of events per covariate would otherwise be below 5. These analyses are descriptive and were not powered for component-specific inference.

CI, confidence interval; eGFR, estimated glomerular filtration rate; HR, hazard ratio; hsCRP, high-sensitivity C-reactive protein; NLR, neutrophil-to-lymphocyte ratio; PIV, pan-immune-inflammation value; SD, standard deviation; SII, systemic immune-inflammation index; SIRI, systemic inflammatory response index.

Table S5. Sensitivity analysis adjusting for troponin T (within-assay-era normal scores).

Panel A. Adjusted hazard ratios for each index with and without troponin T

Index	Clinical model	p	+ admission TnT	p	+ peak TnT	p	Clinical model	p	+ admission TnT	p	+ peak TnT	p
Full 400-day period (n = 2107; 157 events)							***30-day landmark period (n = 2045; 113 events)***					
log(NLR)	1.21 (1.03–1.42)	0.019	1.21 (1.03–1.43)	0.018	1.19 (1.02–1.40)	0.032	1.14 (0.94–1.39)	0.179	1.15 (0.94–1.40)	0.165	1.14 (0.94–1.39)	0.196
log(SIRI)	1.17 (0.99–1.38)	0.058	1.17 (0.99–1.39)	0.057	1.15 (0.98–1.36)	0.093	1.15 (0.95–1.41)	0.160	1.16 (0.95–1.42)	0.145	1.15 (0.94–1.40)	0.177
log(SII)	1.26 (1.07–1.47)	0.005	1.26 (1.07–1.47)	0.004	1.24 (1.06–1.45)	0.008	1.24 (1.02–1.50)	0.030	1.24 (1.02–1.50)	0.028	1.23 (1.02–1.49)	0.033
log(PIV)	1.21 (1.03–1.42)	0.022	1.21 (1.03–1.42)	0.022	1.19 (1.01–1.40)	0.036	1.23 (1.01–1.49)	0.038	1.23 (1.01–1.50)	0.035	1.22 (1.01–1.49)	0.043

Panel B. Independent association of troponin T with MACE in the model containing log(SII)

Period	Troponin variable	HR per 1 SD (95% CI)	p
Full period	Admission troponin T	0.98 (0.78–1.24)	0.865
Full period	Peak troponin T	1.17 (0.91–1.51)	0.228
Landmark	Admission troponin T	0.94 (0.72–1.24)	0.680
Landmark	Peak troponin T	1.04 (0.77–1.41)	0.811

Panel C. Platelet decomposition with troponin adjustment

Period	Adjustment	NLR-based: HR for log(platelet)	LRT p	SIRI-based: HR for log(platelet)	LRT p
Full period	Clinical model	1.13 (0.98–1.31)	0.091	1.12 (0.97–1.30)	0.129
Full period	+ admission troponin T	1.13 (0.98–1.31)	0.094	1.12 (0.97–1.30)	0.132
Full period	+ peak troponin T	1.13 (0.98–1.31)	0.092	1.12 (0.97–1.30)	0.126
Landmark	Clinical model	1.23 (1.03–1.47)	0.020	1.22 (1.02–1.45)	0.028
Landmark	+ admission troponin T	1.23 (1.03–1.46)	0.022	1.21 (1.02–1.45)	0.030
Landmark	+ peak troponin T	1.23 (1.03–1.47)	0.020	1.22 (1.02–1.45)	0.027

The adjusted model included age, sex, hypertension, diabetes mellitus, myocardial infarction presentation, hemoglobin, eGFR, log-transformed hsCRP, multivessel disease and left ventricular ejection fraction. Hazard ratios are expressed per 1-standard-deviation increase in the natural log-transformed index. The troponin T assay changed from a conventional to a high-sensitivity assay in 2018. Values had been converted to a common unit at data entry, but because the analytical sensitivity of the two assays differs at low concentrations, troponin was transformed to van der Waerden normal scores computed separately within each assay era (2012–2017 and 2018–2021), which places the two eras on a common percentile scale; hazard ratios for troponin are therefore per 1 SD of the within-era normal score. Peak troponin T was the highest of the admission and 6–48-hour values. Samples were obtained in the emergency department or at admission, before angiography and before administration of contrast, heparin or antiplatelet loading.

Spearman correlation with peak troponin T: NLR 0.35, SIRI 0.38, SII 0.34, PIV 0.36 and platelet count 0.01; with admission troponin T: 0.43, 0.45, 0.38, 0.40 and -0.05, respectively.

CI, confidence interval; eGFR, estimated glomerular filtration rate; HR, hazard ratio; hsCRP, high-sensitivity C-reactive protein; LRT, likelihood ratio test; MACE, major adverse cardiovascular events; NLR, neutrophil-to-lymphocyte ratio; PIV, pan-immune-inflammation value; SD, standard deviation; SII, systemic immune-inflammation index; SIRI, systemic inflammatory response index; TnT, troponin T.

Table S6. Missing covariate data and multiple-imputation sensitivity analysis.

Panel A. Missing values by covariate (n = 2244)

Covariate	Missing, n	Missing, %
Age	0	0.0
Sex	0	0.0
Hypertension	0	0.0
Diabetes mellitus	0	0.0
MI presentation	0	0.0
Hemoglobin	3	0.1
eGFR	10	0.4
hsCRP	52	2.3
Multivessel disease	0	0.0
LVEF	19	0.8

Panel B. Adjusted hazard ratios: complete-case analysis versus multiple imputation

Index	Complete case (n = 2163) HR (95% CI)	p	Multiple imputation (n = 2244) HR (95% CI)	p	Fraction of missing information
log(NLR)	1.21 (1.04–1.42)	0.017	1.21 (1.04–1.41)	0.014	0.001
log(SIRI)	1.17 (0.99–1.38)	0.059	1.15 (0.98–1.34)	0.087	0.003
log(SII)	1.26 (1.07–1.47)	0.004	1.24 (1.07–1.44)	0.005	0.001
log(PIV)	1.20 (1.03–1.41)	0.024	1.17 (1.00–1.37)	0.044	0.003

Eighty-one patients (3.6%), with 11 events, had at least one missing covariate and were excluded from the complete-case adjusted analyses. Multiple imputation used chained equations (iterative imputation with posterior sampling) with 20 imputed datasets; the imputation model included all covariates, the four indices, platelet count, the event indicator and log follow-up time, and estimates were pooled with Rubin's rules. The adjusted model included age, sex, hypertension, diabetes mellitus, myocardial infarction presentation, hemoglobin, eGFR, log-transformed hsCRP, multivessel disease and left ventricular ejection fraction. Hazard ratios are expressed per 1-standard-deviation increase in the natural log-transformed index.

CI, confidence interval; eGFR, estimated glomerular filtration rate; HR, hazard ratio; hsCRP, high-sensitivity C-reactive protein; LVEF, left ventricular ejection fraction; MI, myocardial infarction.

Table S7. Calibration, overall accuracy and clinical utility of the clinical model with and without each index.

Model	Brier score at 399 d	IPA, %	Integrated Brier score (30–390 d)	Calibration slope (95% CI)	O/E ratio	Net benefit at 7.5% threshold	Net benefit at 10% threshold
Clinical model	0.0689	5.68	0.0413	0.90 (0.74–1.13)	0.98	2.03	1.87
+ log(NLR)	0.0688	5.89	0.0412	0.91 (0.74–1.11)	0.98	2.30	2.03
+ log(SIRI)	0.0689	5.71	0.0413	0.90 (0.73–1.10)	0.98	2.15	2.01
+ log(SII)	0.0687	6.03	0.0412	0.91 (0.74–1.12)	0.98	2.41	1.96
+ log(PIV)	0.0688	5.79	0.0412	0.90 (0.74–1.10)	0.98	2.36	2.01
Treat all	–	–	–	–	–	0.47	-2.30
Treat none	–	–	–	–	–	0.00	0.00

The Brier score at day 399 and the integrated Brier score over 30–390 days were computed with inverse-probability-of-censoring weighting (n = 2163; 159 events). IPA, index of prediction accuracy = 1 − Brier(model)/Brier(null), where the null model is the Kaplan–Meier estimate without covariates (Brier score 0.0731 at day 399). The calibration slope is the bootstrap optimism-corrected slope of the linear predictor (1000 resamples); O/E is the observed Kaplan–Meier 399-day risk divided by the mean predicted risk. Net benefit was computed by decision-curve analysis for survival data (Figure 3) and is expressed per 100 patients; at a threshold probability of 7.5%, the addition of SII to the clinical model corresponded to 0.38 additional net true-positive classifications per 100 patients.

MACE, major adverse cardiovascular events; NLR, neutrophil-to-lymphocyte ratio; O/E, observed-to-expected ratio; PIV, pan-immune-inflammation value; SII, systemic immune-inflammation index; SIRI, systemic inflammatory response index.

Table S8. Period-specific (piecewise-constant) hazard ratios for log(platelet) and log(NLR).

**Period after PCI**	**log(platelet): HR per 1 SD (95% CI)**	**p**	**log(NLR): HR per 1 SD (95% CI)**	**p**
0–30 d	0.97 (0.74–1.27)	0.832	1.48 (1.15–1.91)	0.003
31–90 d	1.66 (1.05–2.61)	0.029	1.21 (0.76–1.92)	0.417
91–180 d	1.35 (0.98–1.86)	0.067	1.09 (0.77–1.52)	0.635
181–400 d	1.06 (0.85–1.31)	0.627	1.10 (0.87–1.39)	0.410
Heterogeneity across periods (3 df)	–	0.137	–	0.305
Linear trend in log(time), event-time split (1 df)	+0.046 (-0.031 to +0.122) per log-day	0.243	-0.043 (-0.116 to +0.030) per log-day	0.247
Scaled Schoenfeld score test, log(time) transform	–	0.109	–	0.989
Scaled Schoenfeld score test, rank transform	–	0.361	–	0.953

Follow-up was split into four episodes (0–30, 31–90, 91–180 and 181–400 days; 44, 15, 31 and 69 events, respectively) and a separate coefficient was estimated for each period in a single Cox model containing the clinical covariates, log(NLR) and log(platelet) (n = 2163). Heterogeneity was tested by likelihood ratio test against the model with a single time-constant coefficient. The linear trend in log(time) was estimated in a model in which follow-up was split at every distinct event time and the product of the covariate with log(time + 1), evaluated at the start of each episode, was entered as a time-varying term, so that the covariate value within a risk set depends only on the current time and never on an individual's own exit time; the scaled Schoenfeld score tests with logarithmic and rank time transforms are the corresponding residual-based tests. See also Figure S1.

CI, confidence interval; HR, hazard ratio; NLR, neutrophil-to-lymphocyte ratio; PCI, percutaneous coronary intervention; SD, standard deviation.

Table S9. Multiplicity-adjusted *p* values for the principal inferential tests.

Test	Raw p	Holm-adjusted p (within family)	Benjamini–Hochberg q (all 21 tests)
Primary association (Table 2, adjusted)			
log(NLR)	0.017	0.051	0.116
log(SIRI)	0.059	0.059	0.140
log(SII)	0.004	0.016	0.084
log(PIV)	0.024	0.051	0.116
Incremental discrimination (Table 3A, ΔC-index)			
log(NLR)	0.110	0.330	0.193
log(SIRI)	0.178	0.330	0.220
log(SII)	0.060	0.240	0.140
log(PIV)	0.110	0.330	0.193
Head-to-head comparison (Table 3B)			
log(NLR)	0.382	0.894	0.401
log(SIRI)	0.298	0.894	0.329
log(PIV)	0.404	0.894	0.404
Platelet decomposition (Tables 3C, 4B)			
full period, NLR-based	0.104	0.208	0.193
full period, SIRI-based	0.146	0.208	0.220
landmark, NLR-based	0.025	0.100	0.116
landmark, SIRI-based	0.033	0.100	0.116
Landmark adjusted association (Table 4A)			
log(NLR)	0.169	0.326	0.220
log(SIRI)	0.163	0.326	0.220
log(SII)	0.030	0.120	0.116
log(PIV)	0.042	0.126	0.126
Platelet-by-period interaction			
NLR-based	0.197	0.334	0.230
SIRI-based	0.167	0.334	0.220

Holm step-down adjustment was applied within each family of related tests; Benjamini–Hochberg false-discovery-rate adjustment was applied across all tests jointly. Bootstrap-based *p* values (incremental discrimination and head-to-head comparison) are from 1000 paired resamples. Only the primary adjusted association of SII with MACE (Table 2) remained significant after within-family Holm adjustment, and no test met the 5% false-discovery-rate threshold when all tests, including the primary analysis, were pooled; every other inferential result in the manuscript should be regarded as exploratory.

MACE, major adverse cardiovascular events; NLR, neutrophil-to-lymphocyte ratio; PIV, pan-immune-inflammation value; SII, systemic immune-inflammation index; SIRI, systemic inflammatory response index.

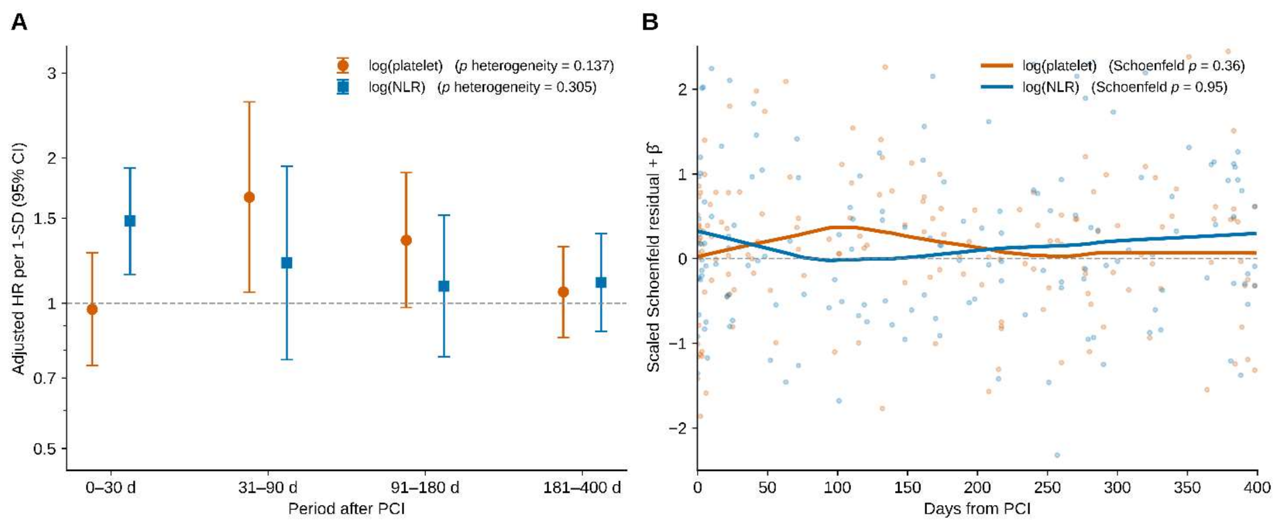

Figure S1. Time-varying effect of platelet count and NLR on 1-year MACE. (**A**) Adjusted hazard ratios per 1-SD for log(platelet) (orange circles) and log(NLR) (blue squares) estimated separately within four periods after PCI, from a single Cox model containing the clinical covariates and both variables; error bars are 95% confidence intervals and *p* values are for heterogeneity across periods (3 degrees of freedom). (**B**) Scaled Schoenfeld residuals plus the time-constant coefficient estimate for the same two variables, with locally weighted regression smooths; *p* values are from the scaled Schoenfeld test of the proportional hazards assumption. Point estimates for NLR were larger in the first 30 days than thereafter, and those for platelet count were near unity in the first 30 days and larger between 31 and 180 days, but neither pattern was statistically significant by the heterogeneity test, by the linear-trend test or by the Schoenfeld test. The p values shown in panel B are from the scaled Schoenfeld score test with rank transformation of time, which is the test of the proportional hazards assumption used throughout the manuscript; the corresponding tests with logarithmic transformation of time, which evaluate a linear trend in log(time) and are reported in Section 3.9 ($p = 0.109$ for platelet count and $p = 0.989$ for NLR), are given in Table S8 together with the rank-transform values.
